# Self‐Assembled Monolayers Facilitate Simultaneous Enhancements of External Quantum Efficiencies and Circularly Polarized Luminescence Dissymmetry Factors for Chiral Perovskite Red Spin‐Light Emitting Diodes

**DOI:** 10.1002/advs.202512057

**Published:** 2025-10-24

**Authors:** Linze Jiang, Guoshuai Zhang, Houzhi Chen, Xiangpeng Zhang, Chao Qian, Jing Li, Lidan Guo, Jinpeng Li, Shuaishuai Ding, Guankui Long, Chuang Zhang, Zhixiang Wei, Xiangnan Sun, Kai Wang

**Affiliations:** ^1^ Key Laboratory of Luminescence and Optical Information Ministry of Education School of Physical Science and Engineering Beijing Jiaotong University Beijing 100044 P. R. China; ^2^ Tangshan Research Institute of Beijing Jiaotong University Tangshan 063000 P. R. China; ^3^ Key Laboratory of Nanosystem and Hierarchical Fabrication National Center for Nanoscience and Technology Beijing 100190 P. R. China; ^4^ Key Laboratory of Photochemical Conversion and Optoelectronic Materials Technical Institute of Physics and Chemistry Chinese Academy of Sciences Beijing 100190 P. R. China; ^5^ State Key Laboratory of Advanced Materials for Intelligent Sensing Tianjin Key Laboratory of Molecular Optoelectronic Sciences Department of Chemistry Institute of Molecular Aggregation Science Tianjin University Tianjin 300072 P. R. China; ^6^ Tianjin Key Lab for Rare Earth Materials and Applications School of Materials Science and Engineering National Institute for Advanced Materials Nankai University Tianjin 300350 P. R. China; ^7^ Key Laboratory of Photochemistry Beijing National Laboratory for Molecular Sciences Institute of Chemistry Chinese Academy of Sciences Beijing 100190 P. R. China

**Keywords:** chiral hybrid perovskites (CHPs), chiral‐induced spin selectivity (CISS), circularly polarized electroluminescence (CPEL), self‐assembled monolayers (SAMs), spin‐light emitting diodes (Spin‐LEDs)

## Abstract

Stemming from the chirality‐induced spin‐orbit coupling (CISOC), the transfer of spin angular momenta to angular momenta of light can be realized by chiral hybrid perovskites (CHPs) for developing novel spin‐light emitting diodes (spin‐LEDs). The primary challenge lies in simultaneous enhancements for the electronic charge associated external quantum efficiencies (EQEs) and spin‐dependent dissymmetry factor of circularly polarized electroluminescence (*g*_EL_), which severely limits the high‐performance spin‐LEDs’ development. Herein, a self‐assembled monolayer (SAM) that acts as an interfacial layer between a nickel oxide (NiO_x_) transport layer and a chiral perovskite emissive film has been adopted for red spin‐LED fabrications. An optimal EQE of 8.3% and *g*_EL_ of 16.3% have been successfully achieved for the red circularly polarized electroluminescence (CPEL) at 725 nm. From the spin‐optoelectronic consideration, the well‐balanced EQE and *g*_EL_. These are attributed to remarkable fluorescence recombination lifetime improvements, ion migration suppressions, and trap density reductions. Notably, a two times greater spin lifetime (≈27.6 ps) has been obtained by comparing with a control sample (≈12.1 ps). Improved chiral‐induced spin‐orbit coupling (CISOC) strengths further elevate spin‐selective capacities, which consequently promote polarized spin current generation. This study unlocks the challenge and opens a new avenue for constructing high‐performance spin‐LEDs using the SAM nanotechnology.

## Graphical abstract

With the self‐assembled monolayer (SAM), external quantum efficiencies and dissymmetry factors for circularly polarized electroluminescence are simultaneously enhanced for chiral perovskite red spin‐light emitting diodes (spin‐LEDs). These are ascribed to the improvements of magneto‐chiroptical and electronic properties. It holds the promise for constructing high‐performance spin‐LEDs using the SAM nanotechnology.

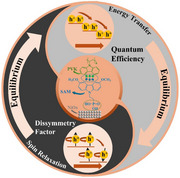

## 1 Introduction

Organic‐inorganic hybrid perovskites (OIHPs) have gained tremendous research and industrial interest for developing high‐performance light‐emitting devices, solar cells, and photodetectors, primarily due to low‐temperature solution‐processibility and large degrees of freedom for tailoring material optoelectronic properties.^[^ ^1^, ^2^, ^3^, ^4^, ^5^ ^]^ Implantations of chiral organic ligands or chiral ionic liquids (CILs) in perovskite octahedral inorganic frameworks extensively lead to the development of chiral hybrid perovskites (CHPs).^[^ ^6^, ^7^, ^8^, ^9^, ^10^ ^]^ It offers a novel pathway for constructing room‐temperature spin‐light emitting diodes (spin‐LEDs), without involving any ferromagnets and magnetic fields.^[^ ^11^, ^12^ ^]^ Over the last five years, the chiral perovskite spin‐LEDs have been fabricated and explored by two main routes: to utilize CHPs as spin injectors, which is analogous to the functionality of ferromagnets;^[^ ^13^, ^14^, ^15^ ^]^ or, to apply them as both spin filters and emissive layers.^[^ ^10^, ^16^, ^17^, ^18^ ^]^ Nevertheless, the main obstacle that hinders their development is to get simultaneous improvements for an external quantum efficiency (EQE) and circularly polarized electroluminescence (CPEL) polarization.^[^ ^19^, ^20^ ^]^

One of the smartest optimization strategies for perovskite growth is to introduce 2D compact self‐assembled monolayers (SAMs). Such monolayers can firmly interact with other adjacent layers in order to realize efficient charge extraction/injection, interfacial energy alignments, and defect passivation. In fact, they have been successfully employed in conventional high‐performance perovskite solar cells and light‐emitting devices.^[^ ^21^, ^22^, ^23^, ^24^ ^]^ Nevertheless, little is known for the impact of SAMs on spin‐optoelectronic properties of CHPs. We may ask: will the rational use of SAMs in the spin‐LEDs offer the opportunity for simultaneously improving EQE and *g*_EL_?

In this work, the SAM, such as MeO‐2PACz is, for the first time, successfully introduced at the interface between a hole transport layer, nickel oxide (NiO_x_), and *p*‐type lead‐iodide based CHPs (i.e., (R‐/S‐p‐F‐MBA)_2_Cs_1_FA_1_Pb_3_I_10_) for fabricating red spin‐LEDs. With the tailored interface, the performance of the spin‐LEDs has been improved remarkably from both electronic charge and spin aspects, which stays currently at the forefront of all the red spin‐LEDs. To the best of our knowledge, this is the first study to demonstrate the impact of SAMs on the magneto‐chiroptical properties of (R‐/S‐p‐F‐MBA)_2_Cs_1_FA_1_Pb_3_I_10_ and spin‐LEDs. There are no additional chiral materials and passivation reagents added into CHPs. Their originally magneto‐chiroptical properties can thus be well‐sustained.

## 2 Results and Discussion

**Figure 1:**
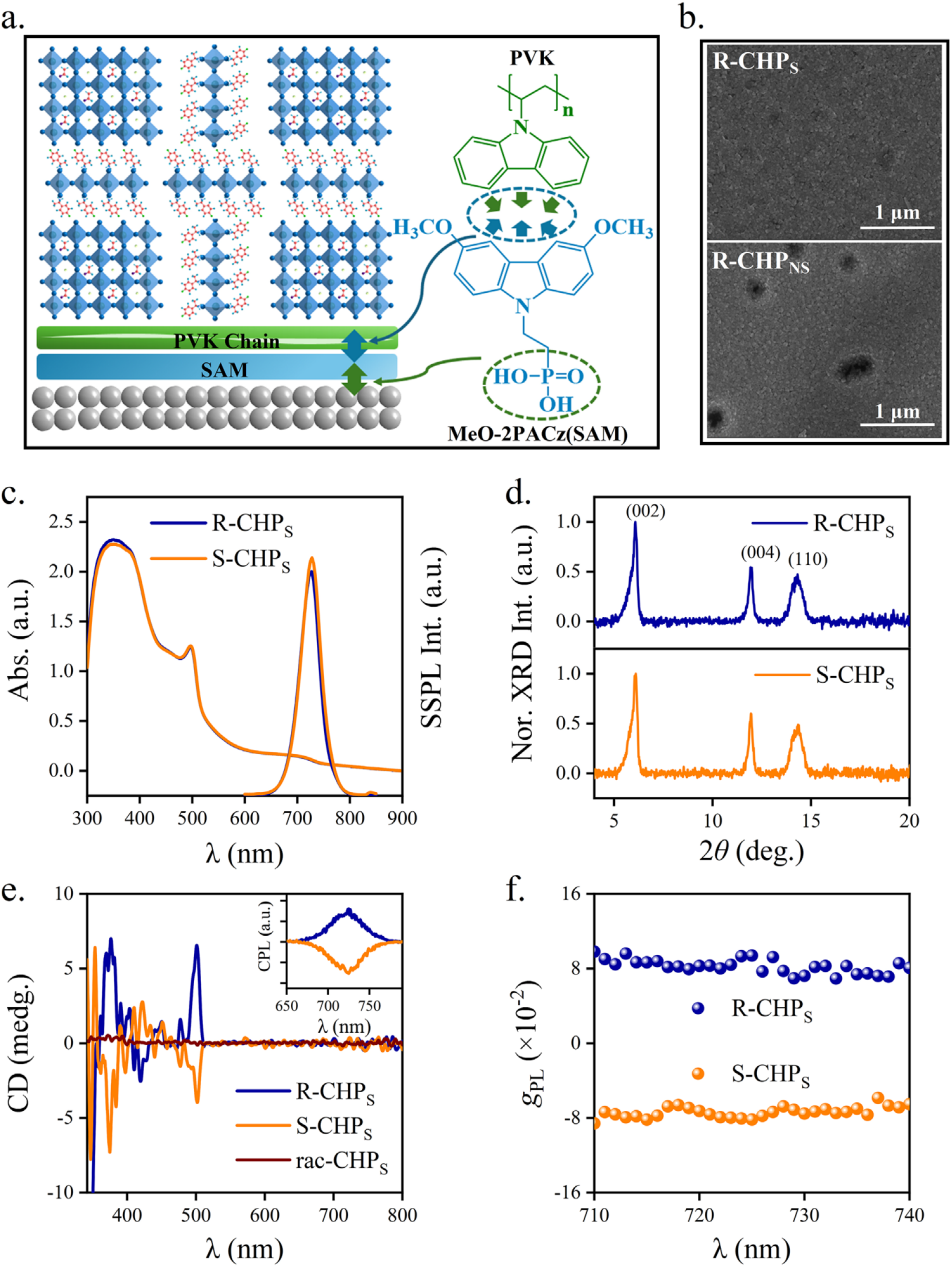
Basic chiroptical and crystalline properties of R‐/S‐CHP_S_. a). The pictorial illustration for the introduction of the SAM layer sandwiched between NiO_x_ and PVK. b). The scanning electron microscopy (SEM) images for the surface morphologies of R‐CHPs with and without using SAM. c). UV–vis absorption spectra and steady state photoluminescence (SSPL) spectra. d). XRD spectra. e). Circular dichroism (CD) spectra, the inset shows circularly polarized luminescence (CPL) spectra. f). Wavelength dependent dissymmetry factor of CPL for R‐/S‐CHP_S_. All the measurements were conducted at room temperature.

In this work, MeO‐2PACz, which is utilized as SAM has the molecular structure in **Figure** **1**a. It is a functional derivative of the sister material 2PACz. The structure of MeO‐2PACz comprises an electron rich 3,6‐methoxylcarbzole unit joined by an anchoring functional group of ethyl phosphonic acid at the 9‐position of the carbazole ring. This allows it to act as an interface between the metal oxides and CHP (Figure 1a). With the assistance of the X‐ray photoemission spectroscopy (XPS) for measuring atomic orbitals, the experimental evidence for validating the growth of SAM on NiO_x_ was given in Figure S1 (Supporting Information). The results clearly indicate the interaction of the chemical constituents between MeO‐2PACz and NiO_x_, consistent with previous studies.^[^ ^25^ ^]^ The details for the fabrication of MeO‐2PACz and CHPs are given in the experimental section. For convenience, R‐/S‐CHP_S_ are used to denote (R‐/S‐p‐F‐MBA)_2_Cs_1_FA_1_Pb_3_I_10_ grown on SAM. As a control experiment, the one without the SAM decoration is denoted by R‐/S‐CHP_NS_. From the observation of the surface morphologies of R‐/S‐CHP_S_ (Figures 1b; S2, Supporting Information), the rational use of the SAM layer results in small and compact crystalline grains with the absence of large surface voids. Figure 1c shows the UV–vis absorption spectra for the polycrystalline films R‐/S‐CHP_S_. The absorption bands at 500 nm stem from the quasi‐2D phase *n* = 1. The absorption band for the large n‐values (≥ 3) appears at 720 nm, coinciding with the steady state photoluminescence (SSPL) peak. The optical energy gap is 1.41 eV. It is observed that to incorporate the SAM layer has no remarkable impact on the lineshape of the absorption spectrum (Figure S3, Supporting Information). Judging from the out‐of‐plane X‐ray diffraction (XRD) spectra in Figure 1d, the diffracted bands that correspond to the 2‐D and 3‐D phases occur at 2𝜃 = 6° and 14°, respectively. They are indexed by the crystalline planes (002) and (110), respectively. Although the XRD spectra of the films R‐/S‐CHP_NS_ have identical diffracted bands (Figure S4 and Table S1, Supporting Information), those grown on top of NiO_x_ without using SAM exhibit relatively reduced diffracted intensities and spectral broadening. The results indicate that SAM helps to increase the crystallinity for the growth of the chiral perovskites. The material chirality is verified by the circular dichroism (CD) spectroscopy, and the results are shown in Figure 1e. The CD spectra indicate the successful chirality transfer by this chiral and nonchiral synergistic method. The racemic film that was fabricated by the same experimental conditions shows the absence of any CD signals. It is noted that the CD signals become weaker for the films R‐/S‐CHP_NS_ with the same film thickness (Figure S5, Supporting Information). The circularly polarized luminescence (CPL) further confirms the presence of the chiral character at the fluorescence peak (≈725 nm, the inset of Figure 1e). The degree of the photoluminescence polarization (DP) is calculated by $\mathrm{DP}\;=\frac{I_{L}^{\mathrm{PL}}-I_{R}^{\mathrm{PL}}}{I_{L}^{\mathrm{PL}}+I_{R}^{\mathrm{PL}}}\;\times 100\%$, where $I_{L}^{\mathrm{PL}}$ and $I_{R}^{\mathrm{PL}}$ are the photoluminescence intensities for the left and right circular polarization, respectively. At λ = 725 nm, DP is 4.7% and 4.1% for R‐CHP_S_ and S‐CHP_S,_ respectively. The corresponding dissymmetry factor of CPL (*g*_PL_) is 9.4% and 8.2% (Figure 1f). For the films R‐/S‐CHP_NS_ fabricated without using SAM, they are reduced to 6.3% and 5.7% respectively (Figure S6, Supporting Information).

**Figure 2:**
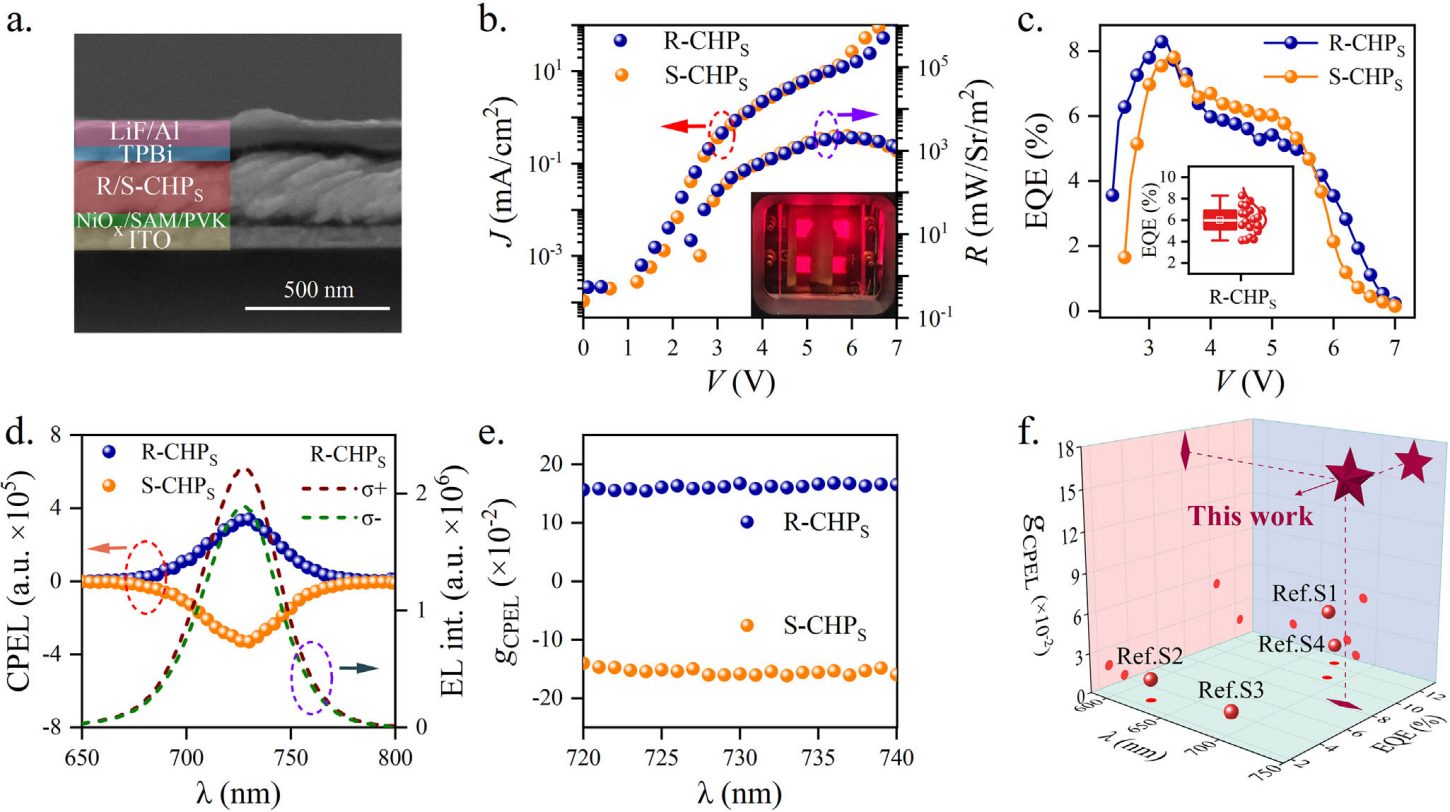
Perovskite spin‐LEDs’ performance. a). The cross‐sectional scanning electron microscopy (SEM) image for the spin‐LED comprising ITO(glass)/NiO_x_/SAM/PVK/R‐CHP/TPBi/LiF/Al. b). The experimental results of current density‐voltage‐luminescence (*J‐R‐V*) characteristic curves for the spin‐LEDs. The inset photographic image displays four spin‐LEDs at working conditions. c). The experimental results of the external quantum efficiency (EQE) for the R‐/S‐CHP based spin‐LEDs. The inset picture shows the statistical summary of EQE. d). The experimental results of the CPEL spectra for the R‐/S‐CHP based spin‐LEDs. 𝜎^+^and 𝜎^−^ denote the left‐ and right‐ circularly polarized light. The inset is the electroluminescence spectra for the R‐CHP based spin‐LED. e). The wavelength‐dependent dissymmetry factor of CPEL for the R‐/S‐CHP based spin‐LEDs. f). Comparisons of different chiral perovskite spin‐LEDs’ performance. Ref.S1: Nano Lett., 24, 6084 (2024); Ref.S2: J. Am. Chem. Soc., 144, 9707 (2022); Ref.S3: Mater. Horiz., 11, 2906 (2024); Ref.S4: Adv. Mater., 37, e2413669 (2025).

From the observation of the cross‐sectional scanning electron microscopic (SEM) image in **Figure** **2**a, the spin‐LEDs have the inverted structure, for instance: ITO(glass)/NiO_x_/SAM/PVK/(R‐/S‐CHP)/TPBi/LiF/Al. The thin films NiO_x_ and PVK serve as the hole transport layers. TPBi acts as the electron transport layer. The ultrathin film LiF is used as the coupling layer to enhance the electron injection. The pictorial illustrations for the spin‐LED structure and the energy levels are given in Figure S7 (Supporting Information). The ladder‐type energy alignments ensure the smooth electrical injection for charge carriers. From the ultraviolet photoelectron spectroscopic (UPS) measurements for NiO_x_ decorated with and without SAM (Figure S8, Supporting Information), to incorporate SAM can further promote hole carrier injection. The experimental results of the current density‐voltage‐radiance (*J*‐*V*‐*R*) measurements are shown in Figure 2b for the spin‐LEDs. The maximum radiance (*R*) is 2073 mW/Sr/m^2^. It is nearly two times greater than the control spin‐LEDs without using SAM (Figure S9, Supporting Information). The turn‐on voltage (𝑉_turn‐on_) is measured to be 2.4 V, which is indeed lower than the control spin‐LEDs as well as most reported spin‐LEDs.^[^ ^14^, ^16^, ^26^, ^27^ ^]^ The inset picture of Figure 2b displays the photographic image for the 2 × 2 spin‐LED matrix at working conditions. The maximum EQE was measured to be 8.3% at *V* = 3.2 V (Figure 2c). For a statistical summary, the reproducibility of EQE for the R‐CHP_S_ based spin‐LEDs is provided in the inset of Figure 2c. In this case, the circularly polarized electroluminescence (CPEL) is characterized by an optical setup including a linear polarizer, a quarter‐wave plate, and a photodetector. The CPEL spectra of the two spin‐LEDs (colored dash lines) are shown in Figure 2d, with the emissive peak at 728 nm. Owing to the chirality, the spectra of R‐/S‐CHP_S_ are mirrored with respect to zero. Analogously with DP, the degree of polarization for CPEL is calculated by $P_{\mathrm{CPEL}}\left(\%\right)=\frac{I_{L}^{EL}-I_{R}^{EL}}{I_{L}^{EL}+I_{R}^{EL}}\;\times 100\%$, where $I_{L}^{EL}$ and $I_{R}^{EL}$ represent the left and right CPEL intensities respectively. At the emissive wavelength of 728 nm, the dissymmetry factor of electroluminescence ( *g*_EL_ =  2 × *P*_CPEL_) is equal to 16.0% for both R‐/S‐CHP_S_ based spin‐LEDs (Figure 2e). For the R‐/S‐CHP_NS_ based spin‐LEDs made without using SAM, they are reduced to 12.3% and 11.9% respectively (Figure S10, Supporting Information). By summarizing all the red spin‐LEDs in the literature, the plot in Figure 2f indicates that ours, due to the application of the SAM nanotechnology, improves EQE, *g*_PL_ and *g*_EL_ simultaneously. The numerical values for all the red spin‐LEDs have detailed in Table S2 (Supporting Information) of the supporting information.

Apparently, in contrast to the control films (i.e., R‐/S‐CHP_NS_) and spin‐LEDs, the SAM method gives rise to the chiroptical properties and the device performance remarkably. The EQE, *g*_PL_, and *g*_EL_ are elevated simultaneously. In the preparation of the chiral perovskite film, the SAM layer behaves like a template for the control of the film morphology and crystallinity. Its dipolar character may also contribute to the charge injection or extraction. Indeed, the film morphology and crystallinity control both electronic and magnetic structures of the chiral perovskite film for photoluminescence and CD.^[^ ^28^ ^]^ In this work, the topology and the crystallinity of the chiral perovskite are changed remarkably due to SAM. Their electronic and magnetic structure can thus be affected. This consequently leads to the change of the dissymmetry g‐factor for the luminescence. In essence, the dissymmetry g‐factor is due to the difference of the left‐ and right‐circularly polarized photoluminescence or electroluminescence, both are highly determined by the material's electronic and magnetic structure. Since the performance of the spin‐LEDs highly relies on the magnetic and electronic properties of R‐/S‐CHP_S_, it thus needs to understand the relevant magneto‐chiroptical properties. From the angle of magnetism, the spin lifetime (τ_spin_), polarized spin current, and chirality‐induced spin‐orbit coupling strength are of critical importance toward the high‐output CPEL.

**Figure 3:**
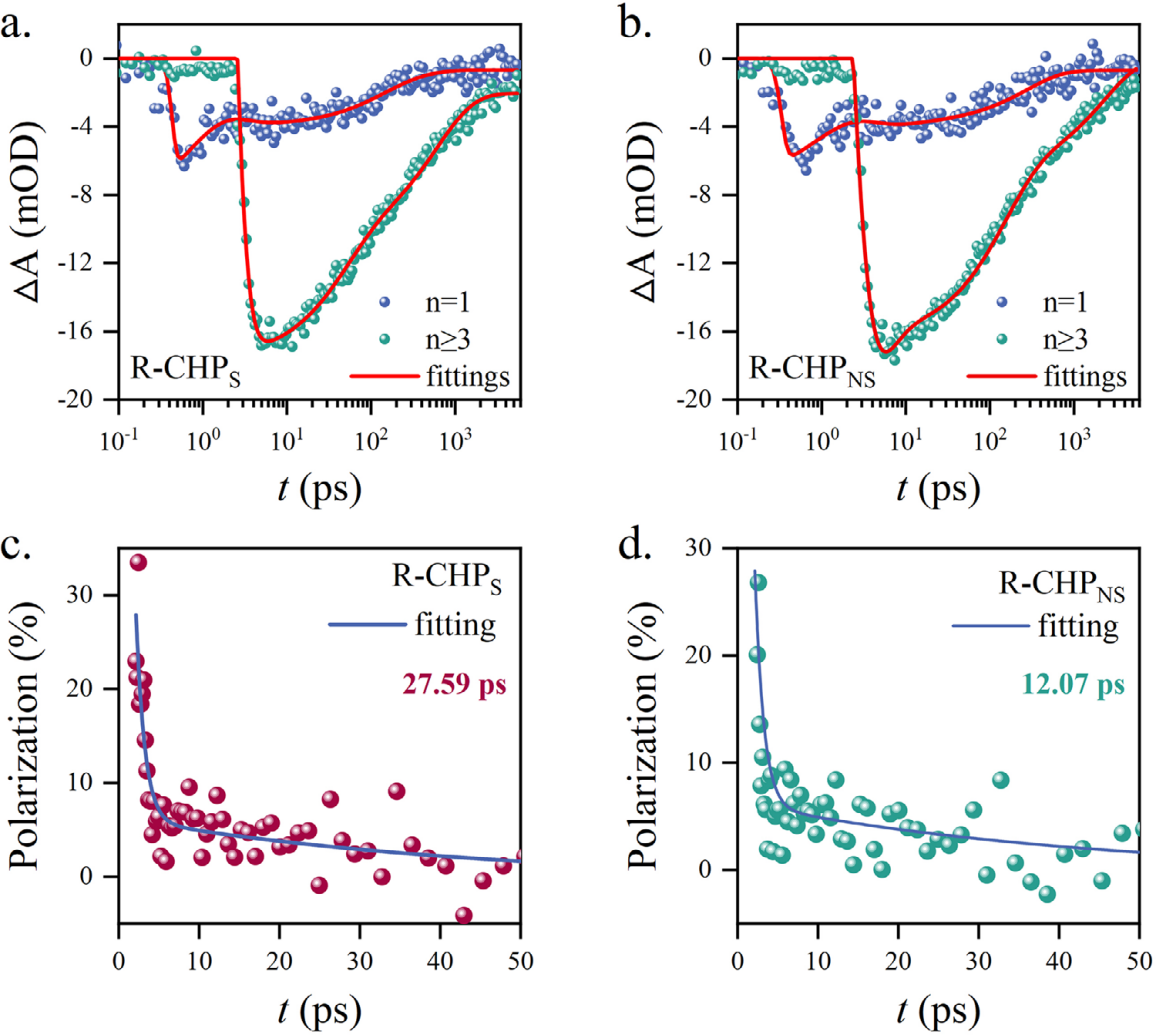
Spin dynamics. a). Transient absorption of CHP films under SAM structure (ITO(glass)/NiO_x_/SAM/PVK/R‐CHP). b). Transient absorption spectra of CHP films without SAM structure. c). Circularly polarized transient absorption (CP‐TA) spectra and spin lifetime of CHP films under SAM structure (ITO(glass)/NiO_x_/SAM/PVK/R‐CHP). d). Circularly polarized transient absorption (CP‐TA) spectra and spin lifetime of CHP films without SAM structure.

In the experiment, the energy transfer and spin dynamics are characterized by the transient absorption (TA) and circularly polarized‐transient absorption (CP‐TA) spectroscopy, respectively. The time evolutions of the TA spectra, which correspond to n = 1 and ≥3 are plotted in **Figure** **3**a,b for R‐CHP_S_ and R‐CHP_NS,_ respectively. The values of the fitting parameters are given in Table S3 (Supporting Information). Judging from the ground state bleach (GSB) absorption at 720 nm, the exciton establishment time (τ_est_) generated by R‐CHP_S_ is 0.64 ps, which is shorter than the one produced by R‐CHP_NS_ (≈1.02 ps). In addition, τ_spin_ is studied by the CP‐TA method. The measurement is based on the manipulation of the polarized pump‐probe beams, so that four possible configurations (σ^+^σ^+^, σ^+^, σ^−^σ^+^, and σ^−^σ^−^) are made. The measuring condition for CP‐TA is fully described in the experimental section. In order to accurately interpret τ_spin_, the average polarization due to CP‐TA (*P*_CP − TA_) over the wavelength 715–725 nm is calculated by $P_{\mathrm{CP}-\mathrm{TA}}=\frac{\Delta A_{\mathrm{counter}}-\Delta A_{\mathrm{co}}}{\Delta A_{\mathrm{counter}}+\Delta A_{\mathrm{co}}}\;\times 100\%$; in which, Δ*A*_counter_ and Δ*A*_co_ represent the absorption differences due to the counter‐ and co‐polarized pump‐probe beams, respectively. After fitting *P*_CP‐TA_ (Figure 3c,d), R‐CHP_S_ has the τ_spin_ of 27.59 ps, which is two times longer than the one without using SAM (≈12.07 ps). By comparing with the aforementioned τ_est_ (≈0.64 ps), the experimental results indicate that the spin coherence can be well preserved upon the energy funnel and carrier transfer from n = 1 to ≥3.

The purpose of using CHPs is to generate polarized carriers based on the material helicity and the CISS effect.^[^ ^29^ ^]^ Such an effect is decided by the so‐called chirality‐induced spin orbit coupling (CISOC), and its strength decides a spin selection capability.^[^ ^30^, ^31^ ^]^ Microscopically, it enables “preferred” spins to participate in electronic transport and luminescence. It also helps to flip “undesirable” spins into “preferred” ones. In the electronic transport, as injected carriers transport through a chiral perovskite with an average velocity $\vec{v}$, they experience a chirality‐related effective magnetic field:

(1) $${\vec{B}}_{\mathrm{chiral}}=-\vec{v}\times \frac{\vec{E}}{c^{2}}=-\frac{1}{qc^{2}}\vec{v}\times \nabla V$$

where, $\vec{E}$ and ∇V denote an internal electric field and potential gradient, respectively. q is the elementary charge. c is the speed of light. The spin‐associated magnetic moment $\vec{\mu }$ can be written by:

(2) $$\vec{\mu }=-g\frac{q}{2m^{*}}\vec{s}=-g\frac{q}{2m^{*}}\frac{\hbar }{2}\vec{\sigma }$$

in which g represents the carriers’ g‐factor, m* is an effective mass, ℏ is the Dirac constant. $\vec{s}$ and $\vec{\sigma }$ denote the spin quantum number and Pauli‐spin matrices, respectively. The combination of the above two equations results in the CISOC Hamiltonian due to the chiral field:

(3) $$H_{\mathrm{CISOC}}=-\vec{\mu }\cdot {\vec{B}}_{\mathrm{chiral}}=-\frac{g\hbar }{4m^{*}c^{2}}\vec{\sigma }\cdot \left(\vec{v}\times \nabla V\right)$$

**Figure 4:**
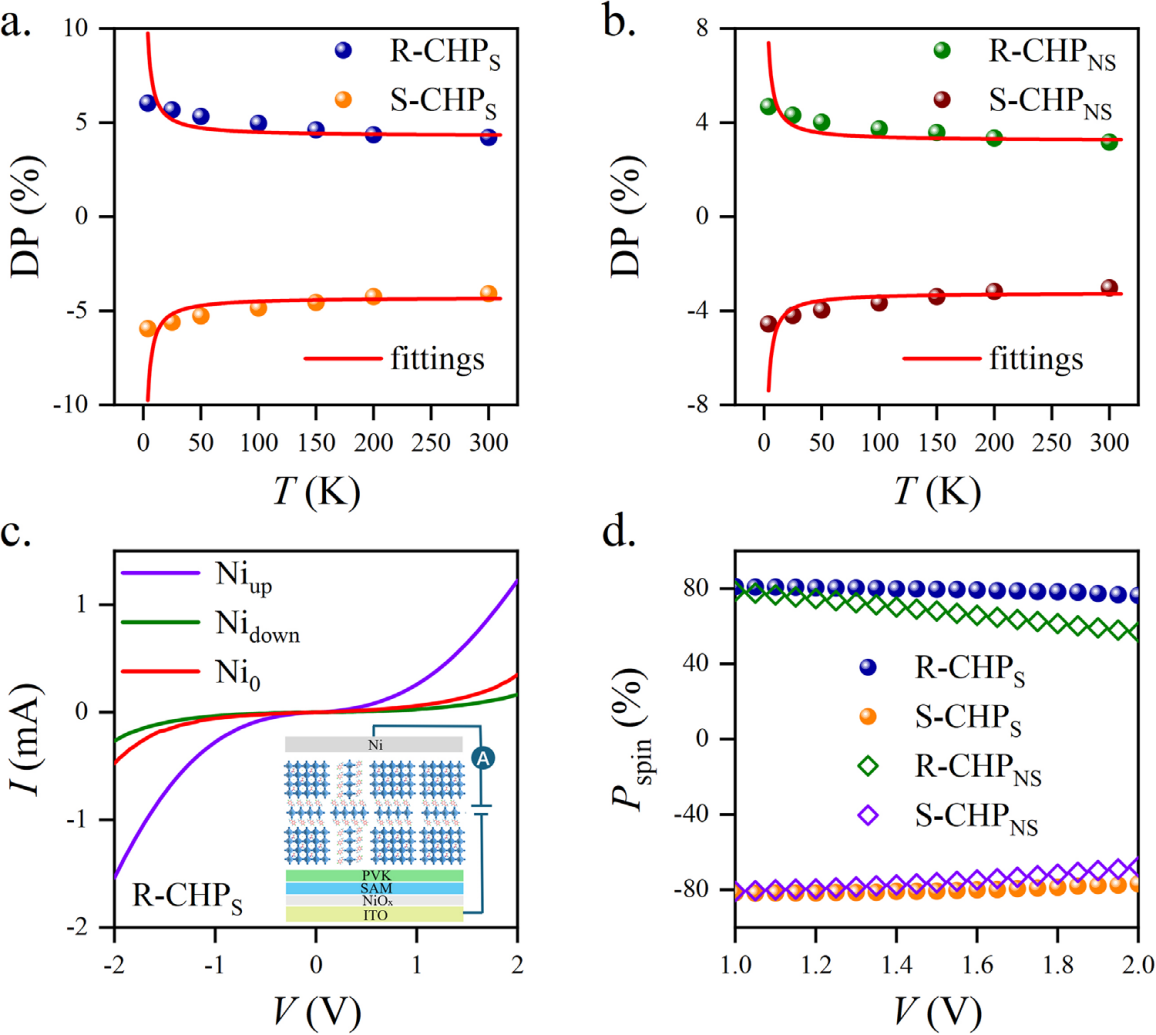
Chirality‐induced spin‐orbit coupling (CISOC) and polarized spin current. The temperature (300–4 K) dependent degree of photoluminescence polarization (DP) due to the circularly polarized luminescence (CPL) for the R‐/S‐CHP films fabricated a). with SAM, and b). without SAM. c). The magneto‐transport measurement for the device consisting of ITO(glass)/NiO_x_/SAM/PVK/CHP/Ni at room temperature. The Ni_up_, Ni_down_ and Ni_0_ represent the magnetization up, down, and zero for the Ni film respectively. d). Bias voltage dependent polarizations for spin currents at room temperature.

In the aspect of CPL, the chirality leads to the spin‐dependent exciton energy band splitting. The light intensities for the left and right circularly polarized light are dictated by occupation numbers for excitons with opposite angular moments at two spin sub‐bands. An energy difference of the two sub‐bands due to CISOC is:

(4) $$\Delta E_{\mathrm{total}}=2\alpha \tau \frac{\sqrt{\varepsilon_{0}}}{c}\omega_{0}+Ak_{b}T$$

in which, *α* denotes the CISOC strength, *τ* = ±1 is the material helicity, *ε*_0_ is the dielectric constant, *ω*_0_ is the photon angular frequency, *A* is the coefficient arising from the thermal perturbation term. *k*_b_ is the Boltzmann constant. *T* is the temperature. In this work, we studied the CISOC strength through the temperature‐dependent DP measurement. The underlying theory has been well documented in our early work.^[^ ^27^ ^]^ As we can see from the experimental results in **Figure** **4**a,b, DP grows steadily as the temperature decreases from 300 to 4 K. The chirality of R‐/S‐CHP_S_ leads to the change of DP in the opposite way. In order to quantify α, the results are modelled by the following expression:

(5) $$\mathrm{DP}\;=\int_{o}^{\pi /2}\frac{cos\theta sin\theta d\theta }{1+cos\theta^{2}}\;\frac{e^{-\frac{2\alpha \tau \frac{\sqrt{\varepsilon_{0}}}{c}\omega_{0}+Ak_{b}T}{k_{b}T}-1}}{e^{-\frac{2\alpha \tau \frac{\sqrt{\varepsilon_{0}}}{c}\omega_{0}+Ak_{b}T}{k_{b}T}+1}}$$

in which *θ* is the angle between the crystal orientation *c*‐axis of the perovskite octahedral inorganic framework and the observing direction. After fittings, α is found to be 0.102 and 0.076 eV·Å for R‐CHP_S_ and R‐CHP_NS,_ respectively. We thus expected the generation of decent polarized spin currents (*P*_spin_) due to the participation of SAM.

In the experiment, *P*_spin_ is measured by the magneto‐transport method for the devices with and without SAM. The devices are fabricated by replacing the stacking layers TPBi/LiF/Al using a soft‐ferromagnetic film Ni of 150 nm thick. Figure 4c shows the asymmetric current–voltage (*I*‐*V*) characteristic curves for the device consisting of ITO(glass)/NiO_x_/SAM/PVK/R‐CHP/Ni when the top Ni film is magnetized by the three different magnetization modes, such as Ni_up_, Ni_down_ and Ni_0_. The degree of polarization for the polarized spin current is calculated by $P_{\mathrm{spin}}=\frac{Ni_{\mathrm{up}}-Ni_{\mathrm{down}}}{Ni_{\mathrm{up}}+Ni_{\mathrm{down}}}\;\times 100\%$. It is found that *P*_spin_ changes from 80% to 75% in the bias window from 1 to 2 V (Figure 4d). The same measurements are performed for the device without using SAM (Figure S11, Supporting Information). In that case, *P*_spin_ changes from 80% to 60% in the same bias window.

**Figure 5:**
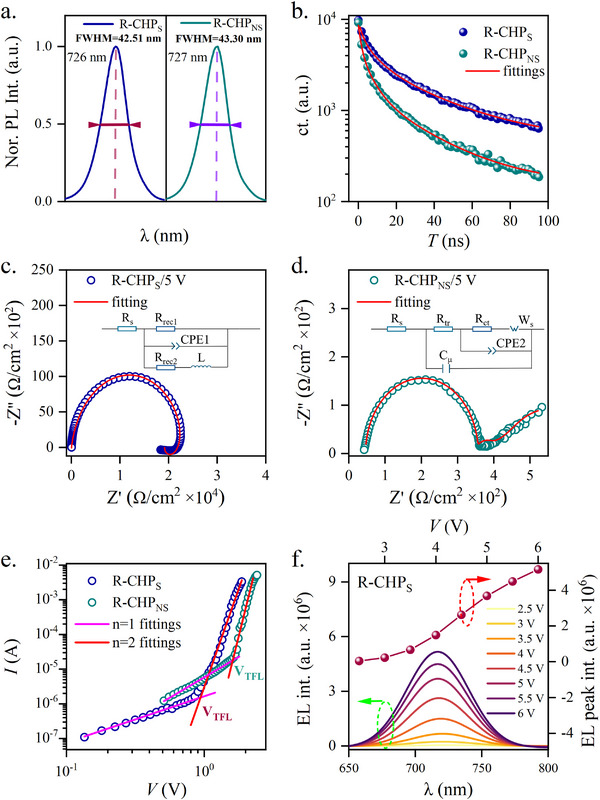
Exciton and electronic charge related properties. a, b). Electroluminescence (EL) and time‐resolved photoluminescence (TRPL) spectra for R‐CHP_S_ and R‐CHP_NS_. c, d). Nyquist plots for the spin‐LEDs with and without using SAM. The inset pictures are the equivalent electronic circuits. In the circuits, *R*_s_ represents the series resistance, *R*_tr_ is the transport resistance, *R*_ct_ is the charge transfer resistance, *R*_rec1_ and *R*_rec2_ are the recombination resistance, CPE1 and CPE2 denote the constant phase elements, L is the inductance, *C*_μ_ represents the chemical capacitance, W_S_ is the Warburg component. e). Dark current–voltage curves of hole‐only devices (ITO(glass)/NiO_x_/SAM/PVK/R‐CHP/MoO_3_/Ag) and (ITO(glass)/NiO_x_/PVK/R‐CHP/MoO_3_/Ag). f). Bias voltage dependent electroluminescence spectra and intensities.

Despite the magnetic properties of R‐/S‐CHPs, the electronic properties are equally important in order to realize the simultaneous enhancement of EQE and the dissymmetry factors. The room temperature electroluminescence (EL) spectrum of the R‐CHP_S_ based spin‐LED shows that the emissive wavelength has a full width at half maximum (FWHM) of 42.5 nm at 726 nm when the bias voltage is 4 V. It is relatively narrower than the one (43.3 nm) produced by the R‐CHP_NS_ based spin‐LED. The reduced FWHM of the EL spectrum is observed for the S‐CHP_S_ based spin‐LED as well (≈41.5 nm, Figure S12, Supporting Information). Judging from the time‐resolved photoluminescence (TRPL) spectra in **Figures** **5**b and S13 (Supporting Information), the fluorescence exciton recombination lifetimes reach 15.44 and 15.35 ns for R‐/S‐CHP_S,_ respectively. They are nearly two times greater than those of R‐/S‐CHP_NS_ (≈7.93 and 7.89 ns). Since the solution‐made CHPs have the ionic crystalline character, the ion migration may be triggered upon the photon or electrical excitation.^[^ ^32^, ^33^ ^]^ It usually has a passive effect on the device performance. In order to understand the ion migration behavior due to the application of the SAM nanotechnology, impedance spectroscopy was performed for the spin‐LEDs in ambient conditions. Figure 5c,d shows the experimental results of the impedance spectra (dotted lines) for the spin‐LEDs with and without using SAM. During the measurements, they stood at the working condition under the application of a constant *dc*‐bias voltage (≈5 V). The Nyquist plots exhibit the different lineshapes so that the distinct equivalent electronic circuits are adopted for the analyses (solid red lines). Such a difference appears in the low‐frequency region, which belongs to the slow time response. Without the SAM decoration, the long and linear tail that is known as the Warburg component indicates the presence of ion migration.^[^ ^34^ ^]^ It disappears for the spin‐LED with the SAM decoration. The results clearly indicate that SAM facilitates the suppression of the ion migration. The same phenomenon can be observed when the *dc*‐bias voltage is set to be 6 V (Figure S14, Supporting Information).

Moreover, the trap density was characterized by the space charge limited current (SCLC) method for the spin‐LEDs with and without using SAM. The spin‐LEDs were made by the structures for instance, ITO(glass)/NiO_x_/SAM/PVK/R‐CHP/MoO_3_/Ag; and, ITO(glass)/NiO_x_/PVK/R‐CHP/MoO_3_/Ag. From the *I*‐*V* characteristic curves (Figure 5e), the trap density was calculated by:

(6) $$n_{t/S\left(NS\right)}=\frac{2\varepsilon_{r}\varepsilon_{0}V_{TFL}}{qL^{2}}\;$$

in which, *n*_*t*/*S*_ and *n*_*t*/*NS*_ denote the trap densities measured with and without SAM. *V_TFL_* is the trap filling limit voltage. *ε*_*r*_ represents the relative dielectric constant (R‐CHP_S_: 7.18; R‐CHP_NS_: 7.12), and they were evaluated by geometric capacitive measurements in Figure S15 (Supporting Information). ε_0_ is the dielectric constant of the free space (≈8.854 × 10^−12 ^F m^−1^). *q* is the elementary charge (*q* = 1.602 × 10^−19 ^C), and *L* is the total thickness of the emissive layer (≈250 nm). Since *V_TFL_* is equal to 0.94 V and 1.64 V respectively, at the intersections of the fitting curves for the ohmic region and the quadratic region in Figure 5e, the corresponding *n*_*t*/*S*_ and *n*_*t*/*NS*_ are therefore equal to 1.19 × 10^16^ and 2.07 × 10^16^ cm^−3^. The stability of the R‐CHP_S_ was examined by the bias voltage‐dependent electroluminescence in ambient conditions. The subsequent increase of the voltage from 2.5 to 6 V leads to the gradual elevation of the electroluminescence intensity (Figure 5f). This step increase in the intensity may also reflect the well‐balanced carrier transport at higher bias voltages. By comparison, the spin‐LED made without SAM exhibits the gradual saturation of the electroluminescence intensity when the applied voltage exceeds 4.5 V (Figure S16, Supporting Information).

## 3 Conclusion

We successfully implemented MeO‐2PACz as SAM for the fabrication of the (R‐/S‐p‐F‐MBA)_2_Cs_1_FA_1_Pb_3_I_10_ based spin‐LEDs. The simultaneous improvements for EQE and *g*_EL_ are realized. With this interfacial engineering method, both electronic spin and charge‐related magneto‐chiroptical properties are studied. This work holds the promise for constructing high‐performance spin‐LEDs using the SAM nanotechnology.

## 4 Experimental Section

### Materials

All the materials are commercially available products. They can be used directly without further purifications. The cesium iodide (CsI, 99.5%), formamidine iodide (FAI, 99.5%), lead iodide (PbI_2_, 99.99%), r‐(+)‐4‐fluorine‐1‐Phenylethylammonium iodide (r‐p‐F‐MBAI, 99.9%), S‐(‐)‐4‐fluorine‐1‐Phenylethylammonium iodide (s‐p‐F‐MBAI, 99.9%), poly(9‐vinylcarbazole) (PVK, average M_n_ ranging from 25 000 to 50 000 g mol^−1^),1,3,5‐tris(1‐phenyl‐1H‐benzimidazol‐2‐yl)benzene (TPBi), and (2‐(3,6‐Dimethoxy‐9H‐carbazol‐9‐yl)ethyl)phosphonic acid (MeO‐2PACz) were purchased from the Xi'an Polymer Light Technology. The nickel oxide (NiO_x_, 99.5%) was purchased from Liaoning Prefers New Energy Technology. The lithium fluoride (LiF, 99.99%) was purchased from Alfa Aesar. The organic solvents N, N‐dimethylformamide (DMF, 99.9%) were bought from Sigma‐Aldrich.

### Spin‐LED Fabrications

The chiral hybrid perovskite (CHP) precursor solutions were prepared by dissolving CsI, FAI, PbI_2_, and R‐/S‐p‐F‐MBAI in the solvent DMF with the molar ratio 1:1:3:2. The R‐/S‐CHP precursor solutions were continuously stirred at room temperature. The ITO‐coated glass (ITO(glass)) substrates were rinsed with deionized water, acetone, and isopropyl alcohol. After this, they were further treated by a plasma oxidation for 10 min. A 15 mg mL^−1^ NiO_x_ solution was prepared by dissolving its powder in deionized water. The solution was placed in an ultrasonic bath for 30 min. It was filtered by a 0.22 µm filter in order to obtain an aqueous NiO_x_ solution. The NiO_x_ transport layer of ≈20 nm was fabricated by the spin‐coating method. The spinning speed and the time duration were set to be 3000 rpm and 30 s, respectively. Then, it was annealed on a hot plate at 120 °C for 10 min. After this, a 0.5 mg mL^−1^ self‐assembled monolayer (MeO‐2PACz) of ≈1.5 nm was spin‐coated on the ITO(glass)/NiO_x_ substrates. The spinning speed and time duration were set to be 2000 rpm and 30 s, respectively. Afterward, all the samples were transferred to an ultrahigh‐purity nitrogen‐filled glovebox. First, the PVK (8 mg mL^−1^ in CB) thin film of ≈30 nm thick was spin‐coated on the ITO(glass)/NiO_x_/SAM substrates with the spinning speed and time duration of 3000 rpm and 30 s. Subsequently, the CHP precursor solution was spin‐coated onto the ITO(glass)/NiO_x_/SAM/PVK at a spin speed of 5000 rpm for 60 s. The sample was annealed at 80 °C for 15 min. This results in a 250 nm film thickness. Finally, the 40 nm thick TPBi, 1.1 nm thick LiF, and 100 nm thick Al were deposited by a thermal evaporation system (base pressure 1 × 10^−5^ Pa). The transport areas were all made to be 2 × 2 mm.

### Material and Device Characterizations

The steady state photoluminescence (SSPL) and time resolved photoluminescence (TRPL) spectroscopy were conducted by a spectrofluorometer (Model: Jobinyvon Horiba Fluorolog‐3). It comprises a continuous‐wave xenon arc lamp, a T‐format sample holder, a monochromator, and a photomultiplier tube. For the TRPL measurement, a 405 nm semiconducting laser diode was used as the photoexcitation source. UV–vis absorption spectra were measured by a standard spectrometer (UV‐2600). Transient absorption (TA) spectroscopy was carried out by using a Helios pump‐probe system (Ultrafast Systems) combined with a regenerative‐amplified Ti: sapphire laser system (Legend Elite‐1K‐HE, 800 nm, 25 fs, 4 mJ pulse^−1^, and 1 kHz repetition rate) and an optical parametric amplifier system. Morphologies of the chiral HOIPs thin films were examined by a field emission scanning electron microscope (SEM, JSM‐6700F) system. Crystalline phases of pristine films were examined by an X‐ray diffraction (XRD) system (Bruker D‐8 Advance). The impedance spectroscopy (Keysight E4990A, 20 Hz–120 MHz) was performed for the Cole‐Cole measurement of the spin‐LED in ambient conditions. During the measurement, a constant *dc*‐bias voltage was applied.

### Magneto‐Chiroptical Characterizations

Circular dichroism (CD) spectra were characterized by a standard circular dichroism spectrometer (Model: Jasco‐1500). Circularly polarized electroluminescence (CPEL) was measured by a lab built‐in electro‐optical system. For the system, a constant dc‐bias voltage or current was applied by a source‐meter unit (Model: Keithley 2400). A quarter‐wave plate (QWP) and a linear polarizer (LP) were placed in between a film/device and a spectrometer. During measurements, LP was kept still while QWP was able to rotate.

### Polarized Spin Current Measurements

Use the chiral LED structure of (ITO(glass)/ NiO_x_/SAM/PVK/(R‐/S‐CHP)/Ni) and (ITO(glass)/NiO_x_/PVK/(R‐/S‐CHP)/Ni). Then the 150 nm thick ferromagnetic Ni layer was evaporated on the chiral perovskite film using an electron beam, with a set‐up speed of 0.5 Å s^−1^. The *I*‐*V* characteristics of the prepared CISS devices were measured using a Keithley 2400 sourcemeter and integrating sphere (Ocean Optics) with the sweeping voltage ranging from −2 to +2 V in dark conditions.

## Conflict of Interest

All the authors declare no conflict of research interest.

## Author Contributions

K.W. conceived the research concept and the experimental designs. K.W., J.P.L., X.S., and Z.W. supervised and guided the whole project. K.W., L.J., and J.P. L. analyzed the data and wrote the manuscript. L.J. performed the fabrication, optimization, and characterization for the chiral perovskites and the spin‐LEDs. G.Z. and C.Q. helped with the device fabrication, optimization, and characterization. X.Z. assisted with the magneto‐transport measurement. K.W. and H.C. performed the theoretical work on the CISOC effect. J.L. helped with the circularly polarized transient absorption spectroscopy. L.G., S.D., G.L., and C.Z. provided fruitful discussions on the results. All the authors finalized the manuscript.

## Data Availability

The data that support the findings of this study are available from the corresponding author upon reasonable request.
